# The association between obesity and social exclusion in middle-aged and older adults: findings from a nationally representative study in Germany

**DOI:** 10.1186/s12877-018-0946-5

**Published:** 2018-10-25

**Authors:** André Hajek, Hans-Helmut König

**Affiliations:** Department of Health Economics and Health Services Research, University Medical Center Hamburg-Eppendorf, Hamburg, Germany

**Keywords:** Body mass index, Obesity, Social isolation, Adult

## Abstract

**Background:**

There is a lack of studies that focus explicitly on the association between *social exclusion* and obesity. The aim of the present study was to identify whether social exclusion is associated with obesity in older adults, and whether this association is moderated by sex.

**Methods:**

Data were derived from wave 5 (2014) of the German Ageing Survey - a representative sample of individuals residing in private households aged 40 and over in Germany. A validated scale developed by Bude and Lantermann was used to assess social exclusion. Individuals with body-mass-index ≥30 kg/m^2^ were classified as being obese. Multiple linear regressions were applied in this study.

**Results:**

Linear regressions showed that social exclusion was not associated with obesity in the total sample and in men, whereas women with obesity were *less* socially excluded than women without obesity (β = −.06, *p* = .02). The corresponding interaction term (sex x obesity) achieved statistical significance (*p* = .03).

**Conclusions:**

Our findings emphasize the negative association between social exclusion and obesity in women, but not men, highlighting the complex interplay between social factors and excess weight in individuals aged 40 and above. Future longitudinal studies are needed to clarify this relationship in further detail.

## Background

Obesity is an important risk factor for mortality and morbidity [1]. Moreover, it is associated with a tremendous financial burden [2]. It has been shown that obesity is highly prevalent in middle aged individuals [3] and individuals aged 75 years and over [4]. It has also been demonstrated that obesity is associated with a decreased quality of life, higher disability, as well as perceived discrimination. These factors can result in feelings of social exclusion. For example, Corica et al. [5] stated that a patient-centered approach that aims at lowering the social exclusion of older adults is required to maintain health-related quality of life in individuals with obesity. Socially excluded individuals can be defined as “people not being involved in different areas of life and their community, eg being unemployed and having a limited social network” [6]. It can lead to distress and anxiety [7].

There are some studies that examine the relation between *loneliness* or *social relationships* and *obesity*. For example, Schumaker et al. [8] found that individuals with obesity had higher loneliness scores than individuals without obesity. Women with obesity scored higher than women without obesity, whereas differences in men with and without obesity were not significant. Moreover, Oliveira et al. [9] examined whether social relationships influenced the incidence of obesity. They found that a lack of emotional support and social relations was associated with an increased incidence of obesity among men. Another study showed that loneliness was positively related to body-mass-index (BMI) values among 182 Spanish women aged 40 to 65 [10]. There are also studies that focus on the relation between social relationships/social support and weight management among middle aged or aged individuals [11–14]. However, there is a lack of studies that explicitly focus on the association between *social exclusion* and obesity. Thus, based on a nationally representative sample among individuals in the second half of life (40 years and over), the aim of the present study was to determine whether social exclusion is associated with obesity in older adults, and whether this association is moderated by sex.

Based on previous findings [8, 9], we hypothesize that individuals with obesity are more socially excluded than individuals without obesity. A possible explanation for this may be that individuals with obesity feel like outsiders [8]. A further explanation may be that they are stigmatized because they do not meet social norms of attractiveness [15]. It is conceivable that obesity impairs social relationships, both in terms of quantity and quality, as well as participation in social activities, because of possible feelings of self-consciousness [8]. As a result, individuals with obesity may feel socially excluded. However, individuals with obesity may befriend other individuals with obesity to better cope with life [8]. Notwithstanding, we assume that these strategies may reduce, but not entirely offset the positive association between social exclusion and obesity, i.e. individuals with obesity may have higher social exclusion scores. Stratified by sex, we hypothesize that the association between obesity and social exclusion is more pronounced in women. Particularly among women, thinness is an important dimension of attractiveness due to, eg, exposure to the media [16]. Hence, it appears plausible that this association is stronger in women than men. Knowledge about an association between social exclusion and obesity, and whether this association is moderated by sex, may be important in assisting individuals at risk for social exclusion.

## Methods

### Sample

For the present study, data from the fifth wave (2014) of the German Ageing Survey (DEAS, “Deutscher Alterssurvey”) were analyzed. The DEAS is a representative cross-sectional and longitudinal survey of the community-dwelling population aged 40 and above in Germany. This survey is organised by the German Centre of Gerontology (DZA, “Deutsches Zentrum für Altersfragen”) and is funded by the Federal Ministry for Family Affairs, Senior Citizens, Women and Youth (BMFSFJ). This survey started in 1996, covering a wide spectrum of topics ranging from social exclusion to the meaning of ageing. Register sampling of the individuals was used, disproportionally stratified by age, gender and location.

The primary inclusion criterion was that individuals had to be 40 years and above. Further inclusion criteria for first time participants were (1) born between 1929 and 1974 and (2) living in private household (which excludes individuals living in institutionalized surroundings). Inclusion criteria for panel participants were (1) one or more valid interviews before (1996, 2002, 2008 or 2011), (2) written consent (willingness to participate in the panel) and (3) still alive as well as not living abroad.

A national representative baseline sample was drawn in the waves 1, 2 (2002), 3 (2008) and 5 and was followed up afterwards. The fourth wave (2011) was a pure panel survey, which means that only individuals who had already taken part before were re-interviewed. In wave 5, while approximately 6000 individuals took part for the first time, more than 4000 individuals were re-interviewed. The response rate was 33%, which is quite similar compared to other large survey studies conducted in Germany [17]. Refusal to participate and bad health condition were main reasons for lack of follow-up data. Among the ‘refusal to participate’ group, a ‘general refusal to participate’ was the main reason. Among this group, other reasons were ‘not interested’ or ‘no time/too long’. Other reasons were negligible. For detailed figures (depending on the subsample), please see [18]. Further details with regards to the DEAS study, such as sample selection or panel mortality, have been provided elsewhere [18, 19], but are also discussed in the discussion section (strengths and limitations) of this paper. Social exclusion was only assessed in the fifth wave. Thus, only data from the fifth wave were used in the current cross-sectional analysis.

The study was conducted according to the principles expressed in the Declaration of Helsinki. Prior to the interview, written informed consent was given. Please note that an ethical statement for this study was not necessary as the criteria for requiring an ethical statement were not met (risk for the respondents, lack of information about the aims of the study, examination of patients). For example, invasive methods were not used.

### Dependent variable

A scale generated by Bude and Lantermann [20] was used to assess social exclusion. It consists of four items (ranging from 1 = “strongly agree” to 4 = “strongly disagree”): “I am worried to be left behind”, “I feel like I do not really belong to society”, “I feel that I am left out”, and “I feel excluded from society”. According to the DEAS guidelines, the scale reflects the mean of at least two required (recoded) valid items. Thus, it was treated as continuous variable, which is in accordance with previous research [21]. Higher values represent higher perceived social exclusion. In our study, Cronbach’s Alpha was .88.

### Independent variables

The variable of interest was the presence of obesity. Height (meter) and weight (kg), both self-reported, were used to calculate BMI (weight divided by height-squared). Individuals with BMI ≥ 30 kg/m^2^ were classified as being obese (0 otherwise). Moreover, potential confounders were identified and controlled for. As socioeconomic covariates, we included age, marital status (married, living together with spouse; others (married, living separated from spouse; single; divorced; widowed), and individual monthly net equivalent income (OECD scale). With respect to lifestyle factors, smoking behavior (daily smoker; casual smoker; former smoker; non-smoker), frequency of sports activities as well as alcohol intake (in both cases, categories were: ‘never’, ‘rarer than once a month’, ‘one to three times a month’, ‘once a week’, ‘several times a week’, and ‘daily’) were identified as possible confounders and controlled for. Furthermore, self-rated health status (from 1 = “very good” to 5 = “very bad”) and the number of chronic illnesses (eg, bad circulation; respiratory problems, asthma, shortness of breath; cancer; diabetes; eye problems, vision impairment; ear problems, hearing problems) were included as covariates.

The covariates were selected based on theoretical assumptions and empirical findings. While the association between obesity and self-rated health as well as chronic illnesses may appear plausible, the other associations may require further explanation. For example, it has been shown that lifestyle factors (smoking behavior, frequency of sports activities and alcohol intake) are strongly associated with obesity [22–25]. Physical activity may also be a factor leading to lower social exclusion, particularly in old age. Moreover, it has been demonstrated that income and educational level are associated with obesity [26]. Since we aimed at identifying the ceteris paribus relationship between obesity and social exclusion, we adjusted for these covariates.

In sensitivity analysis, employment status (employed; retired; other: not employed), and depression (CES-D ≥ 18 [27]) were used.

### Statistical analysis

First, sample characteristics were computed to describe the study participants. Second, multiple linear regressions were used to analyze the association between social exclusion and BMI. Third, because it has been demonstrated that social factors differ between men and women in higher age [28], regressions stratified by gender were conducted. Whether sex moderates the association between social exclusion and BMI was also tested. The statistical significance was determined with *p* < 0.05. Stata 14.0 (StataCorp, College Station, Texas, USA) was used to perform the analyses.

In all regressions, multicollinearity was tested for, using the variance inflation criterion. The largest variance found was 3.16, indicating that a problem with multicollinearity was not present. In addition, the White test for heteroscedasticity in the error distribution was conducted. In all regressions, the test statistics (for example, in the total sample: White’s general test statistic =558.95, *p* < 0.001) lead to the rejection of the null hypothesis of homoscedasticity. Thus, robust standard errors were used in our study.

As there is evidence that perceived weight discrimination differs between obese classes [29], sensitivity analysis was conducted differentiating between these groups (please see the results section for further details).

## Results

### Sample characteristics

Sample characteristics are depicted in Table 1. In sum, 7838 participants (mean age: 64.5 years±11.2 years; 50.4% were aged 65 years and above) provided information about social exclusion. The mean social exclusion score was 1.6 (±0.6; ranging from 1 to 4). Approximately one half were female (51.0%). As for marital status, 70.0% of the individuals were married and living together with spouse. About 21.2% were classified as being obese (BMI ≥ 30 kg/m^2^).

**Table 1:** Sample characteristics (*n* = 7838)

	N/Mean	%/(SD)
Gender: Female	3996	51.0%
Age in years	64.4	11.2
Marital status: married and living together with spouse	5476	70.0%
Monthly net equivalent income in Euro	1943.7	1382.3
Weight classification: Obesity (BMI ≥ 30 kg/m^2^)	1634	21.2%
Smoking status: Daily	1076	13.8%
- Yes, sometimes	309	4.0%
- Not anymore	2879	37.1%
- Never been smoker	3500	45.1%
Consumption of alcohol: Daily	925	12.0%
- several times a week	1882	24.4%
- once a week	1244	16.1%
- one to three times a month	938	12.1%
- less frequently	1862	24.1%
- never	874	11.3%
Physical activity: Daily	663	8.5%
- several times a week	2136	27.3%
- once a week	1427	18.2%
- one to three times a month	592	7.5%
- less frequently	918	11.7%
- never	2101	26.8%
Self-rated health (from 1 = “very good” to 5 = “very bad”)	2.5	0.8
Number of physical illnesses	2.6	1.9
Social exclusion (from 1 to 4; higher values reflect higher social exclusion)	1.6	0.6
Social exclusion: 1	2350	30.0%
> 1 and < 2	2754	35.1%
≥ 3 and < 4	2397	30.6%
≥ 4 and < 5	299	3.8%
5	38	0.5%

Social exclusion was assessed using a scale developed by Bude and Lantermann [20]. Range for number of physical illnesses was 0 to 11

### Regression analysis

The results of the multiple regressions are shown in Table 2 (first column: total sample; second column: men; third column: women). R^2^ values were .11, .13, and .09, respectively. After adjusting for various potential confounders (regression coefficients not shown here, but available upon request), linear regressions showed that while social exclusion was not associated with obesity in the total sample and in men, women with obesity were *less* socially excluded than women without obesity (β = −.06, *p* = .02). In addition, it was tested whether sex moderates the association between social exclusion and BMI. Actually, the corresponding interaction term (sex x obesity) was significant (*p* = .03).

**Table 2:** Determinants of social exclusion (German Ageing Survey, 2014)

	(1)	(2)	(3)
	Social exclusion – Total sample	Social exclusion – Men	Social exclusion - Women
Potential confounders	✓	✓	✓
Obesity (BMI ≥ 30 kg/m^2^; Ref.: Non-obese)	−0.03 (0.02)	0.01 (0.02)	−0.06^*^ (0.03)
Constant	2.50^***^ (0.06)	2.39^***^ (0.08)	2.642^***^ (0.09)
Observations	7041	3532	3509
R^2^	0.11	0.13	0.09

Potential confounders include age, family status, monthly net equivalent income, smoking status, alcohol consumption, frequency of sports activities, self-rated health and number of chronic illnesses. Beta-Coefficients are reported; Cluster-robust standard errors in parentheses. ^***^ *p* < 0.001, ^**^ *p* < 0.01, ^*^ *p* < 0.05, + *p* < 0.10. Observations with missing values were dropped (listwise deletion). Social exclusion was assessed using a scale developed by Bude and Lantermann [20]

In sensitivity analysis (not shown here, but available upon request), survey weights were used in order to ensure the representativeness of the data. Results remained almost the same. For example, women with obesity were less socially excluded than women without obesity (β = −.07, *p* = .03). The interaction term remained significant (*p* = .04).

We also tested whether our findings were dependent on the statistical approach chosen. Therefore, linear regressions were replaced by ordered probit regressions. In terms of significance, our results remained virtually the same.

In further sensitivity analysis, it was tested whether the association between obesity and social exclusion varied by employment status, marital status, depression or age. However, none of the interaction terms achieved statistical significance.

In another sensitivity analysis, the definition of obesity was modified to only include individuals with BMI ≥ 30 kg/m^2^ and BMI < 40 kg/m^2^ in order to exclude Obese Class III individuals, which may strongly affect results. In terms of effect sizes and significance, findings remained virtually the same. In further sensitivity analysis, the definition of non-obesity was restricted to individuals with BMI > 18.5 kg/m^2^ and BMI < 30 kg/m^2^. Again, findings remained almost the same. Thus, findings were not driven by underweight individuals.

In another sensitivity analysis, BMI was classified as: (0) BMI < 30 kg/m^2^, (1) 30 kg/m^2^ ≤ BMI < 40 kg/m^2^ and BMI < 40 kg/m^2^ as well as (2) BMI ≥ 40 kg/m^2^ (63 men; 93 women). When compared with our main models, findings remained almost the same. Women with obesity (Obese class I and II) were less socially excluded than women without obesity (β = −.05, *p* = .04). Women with obesity (Obese class III) were not less socially excluded than women without obesity (β = −.13, *p* = .09).

We also tested whether differences exist between individuals with obesity and (i) individuals with normal weight as well as (ii) overweight individuals. Again, women with obesity were less socially excluded than women with normal weight (β = −.07, *p* = .02). However, women with obesity were *not* less socially excluded than women with overweight (β = −.05, *p* = .06). Apart from that, other significant differences between the groups were not observed.

Stratified by age (younger than 65 years; aged 65 years and above), women with obesity younger than 65 years were *less* socially excluded than women without obesity younger than 65 years (β = −.08, *p* = .01), whereas women with obesity aged 65 years and above were not *less* socially excluded than women without obesity aged 65 years and above (β = −.04, *p* = .35).

## Discussion

### Main findings

Based on a large, population-based sample of individuals in the second half of life, linear regressions showed that social exclusion was not associated with obesity in the total sample and in men, whereas women with obesity were *less* socially excluded than women without obesity, with significant interaction (sex x obesity).

### Relation to previous studies and possible explanations

To date, only a few studies have attempted to determine the association between obesity and factors such as loneliness [8, 9], and there have not been any studies that have sought to determine whether there is an association between obesity and *social exclusion*. We acknowledge that loneliness and social exclusion are correlated. However, they do not measure the same concepts [30]. Individuals feeling lonely may not see themselves as socially excluded and vice versa [31]. These studies have demonstrated that individuals with obesity tend to have higher loneliness scores. Based on these studies, we hypothesized that individuals with obesity have higher social exclusion scores than individuals without obesity. Moreover, it was hypothesized that the association between obesity and social exclusion would be more pronounced in women. However, in contrast to our initial expectations and to previous studies, social exclusion was not associated with obesity in the total sample and men, but there was an association between social exclusion and obesity in women. However, the association in women was in the opposite direction to what was expected. We found that women with obesity were less socially excluded than women without obesity (at least in middle age). Moreover, gender moderates the association between obesity and social exclusion.

In the current study, the non-significant association between social exclusion and obesity among men may be explained by the fact that positive effects (eg, friendship with other individuals with obesity) counterbalance negative effects of obesity (eg, impaired social relationships or feelings of social inhibition). Given the fact that women with obesity face multiple social and economic disadvantages [32], it is somewhat surprising that women with obesity were less socially excluded than women without obesity in our study. A possible explanation for this association may be that women with obesity aged 40 and over have a positive perception of their social relationships. They may feel appreciated by their friends and acquaintances. In other words, a woman with obesity in the second half of life may feel accepted the way she is (regardless of social norms of attractiveness). Furthermore, a higher age and being female were associated with more positive views about obesity in the Swedish general population [33]. Another study has shown that children with obesity in particular, rather than adults or senior citizens are at a higher risk of being confronted with stigmatization [15]. These results [15, 33] may indicate that *older women* with obesity may be more accepted by (female) friends.

It is also conceivable that our findings may be (at least partially) explained by self-selection: Women scoring low in social exclusion are more likely to become obese (affected by unobserved factors such as extraversion). Thus, longitudinal studies investigating the relation between social exclusion and obesity are urgently needed in order to control for time-constant unobserved factors.

### Strengths and limitations

As one of the first studies in this area, we demonstrate that social exclusion and obesity are negatively associated in women, but not men. Data were gathered from a large, population-based sample of individuals in the second half of life. Social exclusion was quantified using a scale with very good psychometric properties. Several potential confounders were used in the analysis. Self-rated BMI was used. It is worth nothing, that as a consequence, it is likely that BMI among the sample was underestimated [34] although this would depend on how the social network affects self-perception of weight. In addition, the caloric intake was not measured in the DEAS study. Furthermore, personality characteristics such as extraversion or openness to experience may be important in the relation between social exclusion and obesity. Self-selection bias may be a concern in the DEAS study. More specifically, it has been shown that participation rates are lower in women, oldest age and individuals residing in large cities [19]. However, it has also been shown that this bias may be relatively small in this survey [19]. Actually, the distribution of socio-demographic attributes (e.g., marital status and educational level) is very close to the distribution to the distribution within the German population [19]. The present study is a cross-sectional one. It could be argued that the causal direction of this relationship (social exclusion and obesity) is bidirectional (development of obesity induced by feelings of loneliness or social exclusion). This is in accordance with the social control theory [35]. Thus, future studies based on longitudinal data are required to clarify this relation.

## Conclusion

Our findings emphasize a negative association between social exclusion and obesity in women, but not men, highlighting the complex interplay between social factors and excess weight in the second half of life. Future longitudinal studies are needed to clarify this relationship in further detail.

## Abbreviations

BMFSFJ: Federal Ministry for Family Affairs, Senior Citizens, Women and Youth
BMI: Body-mass-index
DEAS: German Ageing Survey
DZA: Deutsches Zentrum für Altersfragen
OECD: Organization for Economic Co-Operation and Development
